# Refurbished or Remanufactured?—An Experimental Study on Consumer Choice Behavior

**DOI:** 10.3389/fpsyg.2020.00781

**Published:** 2020-05-19

**Authors:** Yao Chen, Jinfei Wang, Xuening Jia

**Affiliations:** ^1^Management School, Shanghai University of International Business and Economics, Shanghai, China; ^2^Antai College of Economics and Management, Shanghai Jiao Tong University, Shanghai, China

**Keywords:** remanufacturing, purchase behavior/attitude, WTP, product attribute, behavior experiment

## Abstract

Remanufacturing is one of the important means to achieve circular economy and improve the reuse of resources. But, compared with the reuse of old parts, most ordinary Chinese consumers are not familiar with remanufacturing. Because of this, the development of China’s remanufacturing industry is hindered. This paper introduces two kinds of consumer goods with different attributes, namely MP4 (the hedonic product) and cartridge (the functional product). The empirical study on the consumption behavior of Chinese consumers when they are faced with a variety of recycling options was performed. Empirical studies are divided into two stages: participants need to give hypothetical purchase decisions when facing situations of two products (new products and remanufactured products) and three products including refurbishment products, respectively. This paper analyzes the purchase intention and decision-making process of Chinese consumers for remanufactured products, new products, and refurbished products in these two situations. The consumers’ willingness to pay for remanufactured products and refurbished products is also part of the study. The experimental results verify that consumers have a different selection mechanism for new products, remanufactured products, and refurbished products, and there is also a certain relationship between this selection mechanism and the attributes of the product itself. The research shows that due to the different product attributes, consumers pay different attention to the environmental protection, quality, brand, price, and new and old preferences of products. The result of the model shows that the choice behavior of different products and their willingness to pay are also affected by different levels of these attentions. Through the research results, this paper finds conclusions like refurbished products have an impact on the development of remanufactured products, and consumers pay more attention to price but do not pay attention to environmental protection. The conclusion of the study provides references and practical implications for Chinese remanufacturing enterprises to formulate market strategy, for the government to formulate relevant policies, and for OEM production.

## Introduction

In recent years, the concept of circular economy has attracted more and more attention all over the world. The development of remanufacturing industry is one of the important means to realize circular economy and improve the reuse of resources.

**FIGURE 1:**
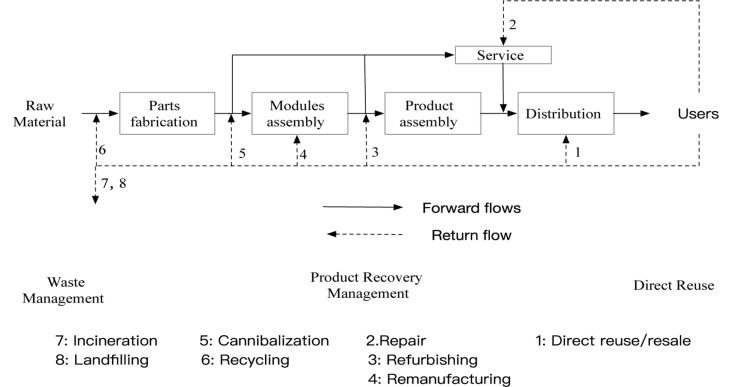
Recycling flow (Ortegon et al., 2014).

According to the definition of product recycling by Thierry et al. (1995), Ortegon et al. (2014), the product recycling method can be divided into five categories: repair, refurbishing, remanufacturing, cannibalization, and recycling (Figure 1) according to the disassembly degree of the product in the process. Among them, the purpose of refurbishment is to recycle the used products and to continue to put them into the market after reaching a certain quality standard, which is lower than the quality standard of the new product; the purpose of remanufacturing is to make the second-hand products meet the same quality standards of the new products before putting them into the market (Chen and Chen, 2019).

Remanufacturing has formed a large-scale industry in developed countries in Europe and the United States, which was first used in the automobile remanufacturing industry. By 2018, the size of the global market has reached about 100 billion United States dollars in annual sales. But in China, the remanufacturing industry started relatively late. In 2017, the output value of China’s auto parts remanufacturing pilot enterprises and related enterprises in the demonstration base exceeded 4 billion yuan, of which the engine remanufacturing output value reached 1.4 billion yuan (Technomic Asia, 2010). For most ordinary Chinese consumers, remanufacturing is a new term, whereas refurbish is a familiar concept. There has been an informal refurbish industry in China for a long time, with a variety of refurbishment machines and parts flooding the market, and China’s remanufacturing industry is facing a more complex competitive environment than that of Europe and the United States. Low-quality refurbished products have occupied the Chinese market for a long time, which is a great obstacle to the promotion of remanufacturing.

For emerging remanufacturing industry in China, the thorniest problem is how to compete more effectively and open the market in the complex market environment. Therefore, with the help of experiments, this paper studies the consumption behavior of consumers when they are faced with the choice of refurbished products and remanufactured goods. Furthermore, it explores the influencing factors of consumers’ willingness to pay (WTP) for remanufactured goods, which has important practical significance for the new remanufacturing enterprises to understand the Chinese market and the competent authorities to formulate relevant industrial policies.

## Literature Review

For more than 20 years, scholars at home and abroad have done a lot of theoretical research on remanufacturing, which can be roughly divided into three aspects: remanufacturing technology, remanufactured product design, and remanufacturing management. In recent years, a group of scholars at home and abroad have studied and explored the problem of remanufacturing market competition. The research team led by Professor Guide of the United States and Professor Wassenhove van of the INSEAD Institute in France is the main research team (Guide and Van Wassenhove, 2003, 2006). Atasu et al. (2008) summarized the latest research progress of this kind of problem.

In the empirical study of remanufacturing market, Camacho-Cuena et al. (2004) and others draw a conclusion through intergroup experiments: underestimating consumers’ WTP will reduce the contribution of recycled products to real WTP. From the point of view of manufacturers, Shu et al. (2019) established a multiremanufacturing model or a two-cycle closed-loop supply chain model based on heterogeneous WTP. The results show that consumers’ high WTP of remanufactured products will make mixed remanufacturing a better choice for manufacturers. Consumers’ WTP for remanufacturing is not only an important factor affecting the development of remanufacturing but also has an important impact on the development of original manufacturers.

There are several aspects concerning consumer preference for remanufactured products.

1. The importance of environmental protection. The experimental results of Michaud and Llerena (2008) showed that the environmental protection information in product characteristics will significantly affect consumers’ environmental preference for remanufactured products. Based on Chinese social survey data by Qin and Song (2019), the premium paid for environmental protection is not very popular, but information about environmental protection can affect consumers’ decision and WTP.
2. The importance of brand. The brand information faced by consumers will affect the purchase choices made by consumers. Subramanian and Subramanyam (2008) use the purchase data on eBay to conclude that a seller’s credit and product category play an important role in the price distinction between new products and remanufactured products.
3. Quality importance. The research results of Abbey et al. (2017) show that consumers’ perception of quality will affect consumers’ preference for remanufactured goods.
4. Price importance. Guide and Li (2010), through eBay online experiments, concluded that consumers are willing to buy remanufactured products at a lower price than new products.
5. The importance of new and old perception. Agrawal et al. (2015) designed consumer choice experiments with MP3 players as stimuli and concluded that consumers’ perceived value of remanufactured products from different sources will affect their perceived value of new products.

In addition, consumers’ awareness of remanufacturing will also affect their purchase decisions.

Liu et al. (2009) conducted a survey of college students to study consumers’ awareness of remanufactured products. The empirical study on the purchase intention of remanufactured goods in Zhang (2011) shows that perceived risk, perceived benefit, and product knowledge are important influencing factors. Sun (2012) experiments show that consumers’ awareness of quality knowledge will significantly affect consumers’ purchase intention. Wang and Hazen (2016) uses intergroup experiments to evaluate consumers’ willingness to buy remanufactured products. The results show that purchase intention is positively affected by perceived value and negatively affected by perceived risk. Through the research on whether the refurbished products are marked with green cycle certification to consumers’ WTP, Harms and Linton (2016) proved that the production of product awareness can improve consumers’ WTP for unfamiliar products significantly. The research of Han and Gu (2016) shows that attitude, internal cognition, and product advantage are the key factors that affect consumers’ purchase intention; trial ability has an important influence on internal cognition and attitude; and the influence of perceived risk on purchase intention is not significant. Xu and Zhu (2017) explored the purchase intention driving mechanism of recycled printing consumables based on marketing strategy by means of a questionnaire. The survey results of Hazen et al. (2017) show that consumers’ attitude toward remanufacturing is an important factor in their decision to buy remanufactured products. Wang et al. (2018) found that for Chinese auto parts remanufacturing consumers, the energy saving, material saving, and emission reduction information of remanufactured products have a positive impact on consumers’ perceived value and trust of remanufactured products.

It can be seen that the existing studies at home and abroad are based on the assumption that remanufacturing is the only recycled product in the market, ignoring the fact that there may be low-quality recycled products in the actual market. In order to better analyze the development dilemma and complex market environment faced by Chinese remanufacturing enterprises, our research team studied the market competition of remanufacturing, renovation, and new products (Chen and Chen, 2019) with a theoretical modeling method on the one hand, and analyzed Chinese consumers’ remanufacturing purchasing behavior and decision-making process with an empirical method on the other hand (Chen et al., 2019). This paper introduces the purchase experiment in our empirical study, examines the process of consumers’ purchase of different products, and studies the influence of refurbished products on the purchase choice of remanufactured products and the influencing factors of consumers’ WTP for remanufactured products.

## Experimental Design

The study of Hirschman and Holbrook (982, 1986) divides the attributes of products and consumers’ attitudes toward consumption into two corresponding dimensions. On the one hand, the hedonistic consumption of the product meets the emotional needs. On the other hand, the functional features of the product meet the functional needs.

MP4 is a kind of product with hedonic properties stronger than functional properties, whereas the cartridge is a printer consumable with stronger functional properties. Both types of products have new products, remanufactured products, and third-party refurbished products in the market. Therefore, the above two stimuli were selected in the study, and the subjects were tested. The following is a simple summary of MP4 as hedonic products and cartridges as functional products.

**FIGURE 2:**
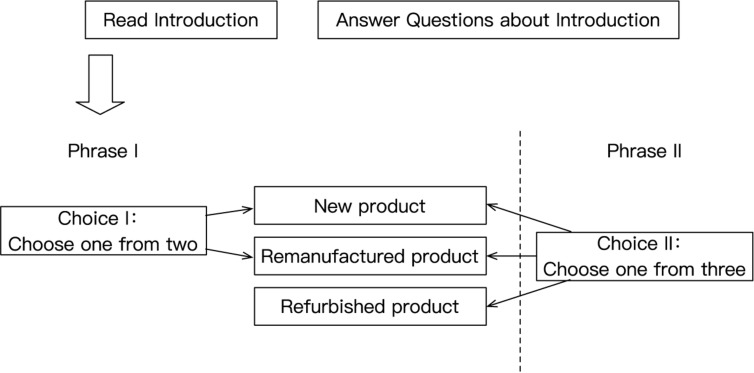
Experimental flow.

The experimental flow is shown in Figure 2. First of all, the subjects of the experiment need to read an introduction to remanufacturing in the experimental environment, and test their understanding and behavioral benchmarks about remanufacturing and refurbished goods through seven questions. Next, subjects need to make two-stage choices for two kinds of stimuli (MP4 and cartridge): in the first stage, subjects are provided with stimuli from different sources for physical and picture display (new products and remanufactured products). After introducing their basic properties to the subjects, make them make the choice of hypothetical purchase in the two products (option I). In the second phase, a third-party refurbished product is introduced on the basis of the first stage. Subjects were also required to make a hypothetical purchase choice among the three products (choose II). After each choice, the subjects need to briefly introduce the reasons for the choice. In addition, for each stimulus, the subjects also need to score the importance of the five features considered in the selection, and give the WTP and quality estimation of the remanufactured and refurbished product.

Among them, 537 subjects participated in our experiment, and the proportion of the participants in the 20-year-old age group and 30-year-old age group is about 51.87% and 33.33%, respectively; the proportion of the subjects with a bachelor’s degree is 54.61%, whereas those with a master’s degree or above is 32.26%. The ratio of men to women is 4:6. The proportion of people aged between 20 and 30 is about 51.87%, and aged between 30 and 40 is about 33.33%. The major backgrounds of the participants include economics, management, software, power engineering, law, mathematics, English and art, etc. The span of the major and age reduces the impact of the background on the results. All the answers in this experiment were conducted anonymously.

This paper puts forward the following hypothesis. First of all, for the choice of consumer products, consider whether there are two kinds of markets for refurbished products, and put forward the following hypothesis:

In addition, with regard to the motivation of consumers’ WTP for remanufacturing, taking into account the market in which refurbished products exist, respectively put forward the following hypothesis:

## Experimental Results

### Variable Description

Through the references, nine representative explanatory variables are selected in the experiment, as shown in Table 3.

**TABLE 1:** Hypothesis of consumer choice motivation.

**Situation**	**Hypothesis**	**Hedonistic product**	**Functional product**
No refurbished	Customers who attach importance to price may prefer remanufactured products to new ones.	H1a1	H1a2
product	Customers who attach importance to environmental protection may prefer remanufactured products to new ones.	H1b1	H1b2
Refurbished	Customers who attach importance to price may prefer new products to remanufactured ones.	H2a1	H2a2
product exists	Customers who attach importance to price may prefer refurbished products to remanufactured ones.	H2b1	H2b2
	Customers who attach importance to environmental protection prefer remanufactured products to new ones.	H2c1	H2c2

**TABLE 2:** Hypothesis of consumers’ WTP.

**Hypothesis**	**Hedonistic product**	**Functional product**
The more consumers pay attention to environmental protection, the higher the WTP for remanufactured goods.	H3a1	H3a2
The more consumers attach importance to quality, the lower their WTP for remanufactured goods.	H3b1	H3b2
The more consumers attach importance to the brand, the lower the WTP for remanufactured goods.	H3c1	H3c2
The more consumers pay attention to the price, the higher the WTP for remanufactured goods.	H3d1	H3d2
The more consumers attach importance to the old and the new, the lower the WTP for remanufactured goods.	H3e1	H3e2
The higher consumers’ awareness of remanufacturing, the higher their WTP for remanufactured goods.	H4a1	H4a2
The higher the level of consumers’ awareness of new products, the higher their WTP for remanufactured products.	H4b1	H4b2
The richer the consumer’s purchase experience of remanufactured goods, the higher the WTP for remanufactured goods.	H4c1	H4c2
The richer the consumer’s purchase experience of remanufactured goods, the higher the WTP for remanufactured goods.	H4d1	H4d2

**TABLE 3:** Variable definition and description of statistical results.

**Variables**	**Meaning**	**Related literature**	**Mean**		**SD**	
			**MP4**	**Cartridges**	**MP4**	**Cartridges**
WTP_*M*_	WTP for remanufactured products	Camacho-Cuena et al., 2004; Chen and Chen, 2019	δ = 0.45	δ = 0.44	0.21	0.18
WTP_*F*_	WTP for refurbished products		δ = 0.21	δ = 0.23	0.20	0.19
Q_*M*_	Estimated quality for remanufactured products	Grewal et al., 1998; García-Fernández et al., 2018	0.77	0.73	0.21	0.18
Q_*F*_	Estimated quality for refurbished products		0.55	0.50	0.24	0.25
E	Environmental protection importance	Michaud and Llerena, 2008; Qin and Song, 2019	2.14	2.41	1.22	1.29
Q	Quality importance	Abbey et al., 2017; Zhao and Zheng, 2013; Abbey et al., 2019	5.37	5.37	0.96	0.97
B	Brand importance	Subramanian and Subramanyam, 2008	3.96	3.70	1.37	1.36
P	Price importance	Taylor and Neslin, 2005	4.21	4.58	1.27	1.18
N	New and old importance	Agrawal et al., 2015; Essoussi and Linton, 2010	3.07	2.73	1.51	1.42
Know_*M*_	Knowledge level for remanufactured products	Sun, 2012	1.88		0.565	
Know_*F*_	Knowledge level for refurbished products		2.08		0.546	
Expe_*M*_	Purchasing experience for remanufactured products	Howard and Sheth, 1969	1.42		0.695	
Expe_*F*_	Purchasing experience for refurbished products		1.7		0.763	

The subjects’ WTP for remanufactured products is quite different from that for remanufactured products, which is correct for both MP4 and cartridges. In terms of the importance of the five major decision-making factors, the subjects paid the most attention to quality, followed by price, old and new perception, brand, and environmental protection.

### The Influence of Refurbished Products on Remanufacturing Consumers

Observing the existence of two informal third-party new products to stimulants will affect the market of new products and remanufactured products to a certain extent, but the impact on the two types of goods is obviously different (see Tables 4a and 4b). Then, statistics are made on the selection of new products, new products, or insistence on remanufactured products among the subjects who choose remanufactured products in the first stage.

**TABLE 4a:** Consumers’ choice of MP4 I and II (%).

**Percentage**		**Choice II**			**Total**
		**New product**	**Remanufactured product**	**Refurbished product**	
Choice I	New product	51.02	2.61	8.75	62.38
	Remanufactured product	0.56	28.68	8.38	37.62
Total		51.6	51.58	31.28	17.13

**TABLE 4b:** Consumers’ choice of cartridges I and II (%).

**Percentage**		**Choice II**			**Total**
		**New product**	**Remanufactured product**	**Refurbished product**	
Choice I	New product	22.99	3.36	15.70	42.06
	Remanufactured product	1.87	35.14	20.93	57.94
Total		24.86	38.50	36.64	100.00

Among the 202 subjects who chose to remanufacture MP4 in the first stage (accounting for 37.62% of the total), 22.28% chose new products in II, 1.49% chose new products in II, and the remaining 76.24% insisted on remanufactured products (Table 5a) (Supplementary Datasheet S1). Among the 310 subjects who chose to remanufacture the cartridge in the first stage (accounting for 57.94% of the total), 36.13% chose the new product in the choice of II, 3.23% chose the new product in the choice of II, and the remaining 60.65% insisted on choosing the remanufactured product (Table 5b).

**TABLE 5a:** Statistics of selection change of remanufactured MP4 caused by refurbishment.

**Change of choice**		***N***	**Percentage**
Selected subjects selected for remanufacturing of MP4 on Stage 1	Choice changed into new MP4	3	1.49
	Insist on choosing remanufactured MP4	154	76.24
	Choice changed into refurbished MP4	45	22.28
	Total	202	100

**TABLE 5b:** Statistics of selection change of remanufactured cartridges caused by refurbishment.

**Change of choice**		***N***	**Percentage**
Selected subjects selected for remanufacturing of cartridges on Stage 1	Choice changed into new cartridges	10	3.23
	Insist on choosing remanufactured cartridges	188	60.65
	Choice changed into refurbished cartridges	112	36.13
	Total	310	100

**FIGURE 3:**
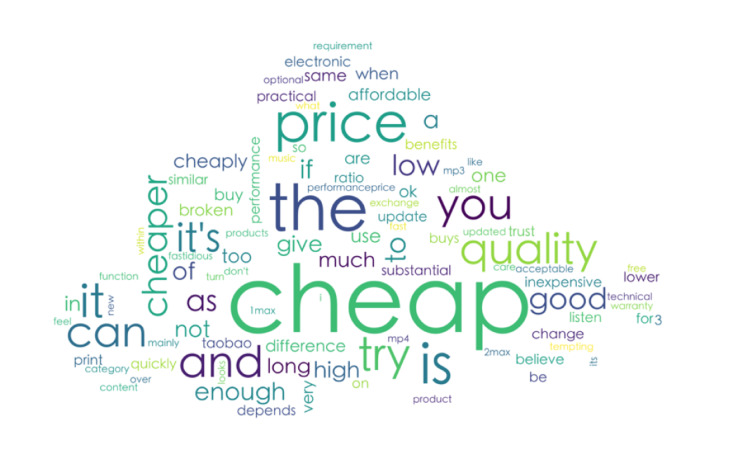
Reasons for choosing refurbished products.

According to the analysis of the reasons for the selection and purchase of the subjects, the people who chose the refurbished products were mainly attracted by the low price of the refurbished products and belonged to the price-sensitive consumers (see Figure 3).

### Logit Regression Analysis

Taking the subjects who chose remanufactured products as survey samples, the Logit model was used to compare the subjects’ two different attitudes of “insisting on choosing remanufactured products” and “abandoning remanufactured products and choosing new products.” The results of Tables 6a and 6b show that the L_1__*a*_ and L_1__*b*_ models are as follows:

**TABLE 6a:** L_1_ logit Regression Analysis (changes in purchase options for MP4).

**Choice changed into refurbished MP4**		***B***	**SE**	**Sig.**	**Exp (*B*)**
Stage 3 a	Estimated the quality of remanufactured MP4	−6.223	1.483	0.000	0.002
	Estimated refurbished MP4 quality.	3.797	1.134	0.001	44.576
	Price importance	0.75	0.215	0.000	2.118
	Constant	−2.069	1.404	0.141	0.126

*a. The reference category is: Insist on choosing to remanufacture MP4.*

**TABLE 6b:** L_1_ logit Regression Analysis (changes in purchase options for cartridges).

**Choice changed into refurbished cartridges**		***B***	**SE**	**Sig.**	**Exp (*B*)**
Stage 3 a	Estimated the quality of remanufactured cartridges	−2.34	1.029	0.023	0.096
	Estimated refurbished cartridges quality.	3.474	0.821	0.000	32.271
	Quality importance	−0.315	0.137	0.022	0.73
	Price importance	0.438	0.159	0.006	1.549
	Constant	−1.047	1.348	0.437	0.351

*a. The reference category is: Insist on choosing to remanufacture cartridges.*

$$=-2.069-6.223\,Q_{M}+3.797\,Q_{F}+0.75\,P$$

$$\exp(\mathrm{Give}\mathrm{up}\mathrm{remanufactured}\mathrm{products}\mathrm{Logit}\,\,\left[\frac{\mathrm{and}\mathrm{choose}\mathrm{new}\mathrm{cartridges})}{\exp\,\left(\mathrm{Insist}\,\mathrm{on}\,\mathrm{choosing}\,\mathrm{remanufactured}\,\mathrm{cartridges}\right)}\,\right]$$

$$=-1.047-2.34\,Q_{M}+3.474\,Q_{F}-0.315\,Q+0.438\,P$$

There are three variables that enter both two models. They are the estimated quality for remanufactured and refurbished products, as well as the importance of price. Through the analysis of the regression results, the following conclusions can be drawn:

1. The higher the estimation of the quality of remanufactured products, the higher the probability of insisting on buying remanufactured products, and the longer the estimated duration of remanufactured products, the lower the probability of insisting on buying remanufactured products.
2. Price is the main competition point for remanufactured products and new products, and subjects who are price-sensitive are more likely to be attracted by refurbished products.
3. For cartridges, the degree of attention to quality is also one of the significant factors affecting whether consumers change their choices. The more consumers pay attention to quality, the more likely they are to simply buy remanufactured cartridges.

### Influencing Factors of Consumers’ Choice in Two Kinds of Market Conditions

Logit regression is constructed for consumer choice in two markets (with or without refurbished products), and the results are shown in Tables 7a and 7b.

**TABLE 7a:** Logit regression result of Model I, II (MP4, hedonistic products).

**Explained variable**		**Model I**		**Model II**	
		**$L\,o\,g\,i\,t\,\left(\frac{e\,x\,p\,\left(\text{Rema}\right)}{e\,x\,p\,\left(N\,e\,w\right)}\right)$**	**$L\,o\,g\,i\,t\,\left(\frac{e\,x\,p\,\left(R\,e\,m\,a\right)}{e\,x\,p\,\left(\text{New}\right)}\right)$**	**$L\,o\,g\,i\,t\,\left(\frac{e\,x\,p\,\left(\text{Refu}\right)}{e\,x\,p\,\left(\text{New}\right)}\right)$**	**$L\,o\,g\,i\,t\,\left(\frac{e\,x\,p\,\left(R\,e\,m\,a\right)}{e\,x\,p\,\left(R\,e\,f\,u\right)}\right)$**
Independent variable	Willingness to pay for remanufactured MP4	5.42***	6.217***	1.387^†^	4.831***
	Willingness to pay for refurbished MP4	−0.816	0.247	3.243***	−2.995***
	Environmental protection importance	0.107	0.1	−0.19	0.29*
	Quality importance	−0.428***	−0.515***	−0.683***	0.167
	Brand importance	−0.217**	−0.166^†^	−0.321**	0.155
	Price importance	0.136^†^	0.104	0.688***	−0.583***
	New and old importance	−0.189**	−0.338***	−0.259**	−0.08
Reference variable		New product	New product	New product	Refurbished product

^†^*p* < 0.1, *P < 0.05, **P < 0.01, ***P < 0.001.

**TABLE 7b:** Logit regression result of Model I, II (Cartridge, functional products).

**Explained variable**		**Model I**		**Model II**	
		**$\text{Logit}\,\left(\frac{\exp\left(R\,e\,m\,a\right)}{\exp\left(N\,e\,w\right)}\right)$**	**$L\,o\,g\,i\,t\,\left(\frac{e\,x\,p\,\left(R\,e\,m\,a\right)}{e\,x\,p\,\left(\text{New}\right)}\right)$**	**$L\,o\,g\,i\,t\,\left(\frac{e\,x\,p\,\left(\text{Refu}\right)}{e\,x\,p\,\left(\text{New}\right)}\right)$**	**$L\,o\,g\,i\,t\,\left(\frac{e\,x\,p\,\left(R\,e\,m\,a\right)}{e\,x\,p\,\left(R\,e\,f\,u\right)}\right)$**
Independent variable	Willingness to pay for remanufactured MP4	6.926***	6.366***	1.446^†^	4.921***
	Willingness to pay for refurbished MP4	−1.403*	−2.651**	2.116*	−4.767***
	Environmental protection importance	0.132	0.07	0.093	−0.024
	Quality importance	−0.64***	−0.43***	−0.504***	0.074
	Brand importance	−0.39***	−0.375***	−0.286**	−0.089
	Price importance	0.573***	0.566***	0.766***	−0.2*
	New and old importance	−0.134^†^	−0.208*	−0.169^†^	−0.039
Reference variable		New product	New product	New product	Refurbished product

^†^*p* < 0.1, *P < 0.05, **P < 0.01, ***P < 0.001.

The results of model I in Table 7a show that when there are only two purchase options: new product MP4 and remanufactured MP4, the higher the WTP for remanufactured MP4, the easier it is to choose remanufactured MP4. Quality, brand, new and old perception, and price attention also have a significant impact on the subjects’ purchase choices. The subjects who pay more attention to quality, brand, or the perception of old and new are more unwilling to choose to remanufacture MP4, and the more they pay attention to price, the more likely they are to choose to remanufacture MP4. At the same time, the absolute value of the regression coefficient of the quality factor is greater than the price factor, indicating that the importance of quality is generally greater than the price. The results of model I in Table 7b show that in addition to the accident of WTP, the importance of quality, brand, and price has a significant impact on the subjects’ purchase choices. Consumers who pay more attention to quality, brand, and perception of old and new are more likely to choose new products, whereas consumers who pay attention to price are more likely to prefer remanufactured products. Therefore, hypothetical H1a1 and hypothetical H1a2 are confirmed, but H1b1 and H1b2 cannot be confirmed.

The result of model II shows that when facing the three choices of new product, remanufactured product, and refurbished product, the decision-making process of the subjects becomes more complicated than that of choosing one of the two. From Table 7a, it can be found that due to the intervention of refurbished products, subjects play a greater role in the WTP for remanufactured MP4 when measuring new and remanufactured products, the factors of quality and perceived importance between new and old become larger, the factors of brand become smaller, whereas the price factor is not significant. Table 7a has similar results, but price is still one of the decision-making factors that significantly affect consumers’ purchase and remanufactured cartridges. Therefore, it is assumed that H2a1 is not proved and H2a2 is proved.

The entry of refurbished products into the market also has a great impact on new products, and almost all the entry variables are significant in this model. For MP4 and cartridges, price attention has become the most influential factor besides the accident of WTP on customers’ choice of new products and new products. In the comparison of remanufactured products and refurbished products, the subjects’ choice of remanufactured products not only is affected by the WTP but also plays a significant role in price attention. Among them, consumers will also receive a significant impact on the importance of environmental protection when they choose MP4. Consumers who pay more attention to price are more likely to choose new products, whereas consumers who pay more attention to environmental protection are more likely to choose to reproduce MP4. It is worth mentioning that in the environment where the concept of hedonism is strong and the awareness of environmental protection is generally not high, this is not conducive to consumers giving up refurbished products and choosing to remanufacture products. This is especially true for products with strong hedonistic properties. Therefore, H2a1, H2a2, and H2c2 were not confirmed, whereas H2b1, H2b2, and H2c1 were confirmed. The hypothetical results about consumers’ motivation for choice are shown in Table 8.

**TABLE 8:** Consumer choice motivation hypothesis.

**Situation**	**Hypothesis**	**Hedonistic product**		**Functional product**	
No refurbished product	Customers who attach importance to price may prefer remanufactured products to new ones.	H1a1	✓	H1b	✓
	Customers who attach importance to environmental protection may prefer remanufactured products to new ones.	H1b1	×	H2b	×
Refurbished product exists	Customers who attach importance to price may prefer new products to remanufactured ones.	H2a1	×	H3b	×
	Customers who attach importance to price may prefer refurbished products to remanufactured ones.	H2b1	✓	H4b	✓
	Customers who attach importance to environmental protection prefer remanufactured products to new ones.	H2c1	✓	H5b	×

### Analysis on the Motivation of the Purchase of Remanufactured Goods

In this section, the linear regression method will be used for analysis, taking into account the factors affecting the WTP of remanufactured goods in the market where remanufactured goods exist (see Table 9). Models M1 and M2 take the WTP of remanufactured MP4 as dependent variables. Before and after adding the control variable, the price emphasis and the perceived importance of new and old have a significant impact on the remanufacturing WTP. The higher the price attention, the lower the perceived attention of new and old, and the higher the WTP of remanufactured MP4. As far as the WTP of remanufacturing toner cartridges is concerned, the importance of environmental protection and price is significant in models M5 and M6. The consumers who pay more attention to environmental protection and price are more willing to pay for remanufactured cartridges. For two different products, the price emphasis is significant in the four models, whereas for hedonic products such as MP4, consumers’ perceived attention to new and old has a more significant impact on their WTP; for functional products such as cartridges, consumers’ awareness of environmental protection will have a more significant impact on their WTP.

**TABLE 9:** Regression coefficient (Remanufactured MP4 willingness to pay).

	**M1**	**M2**	**M3**	**M4**	**M5**	**M6**	**M7**	**M8**
Constant	0.3*	0.385*	0.349*	0.364*	0.21	0.292^†^	0.406**	0.425**
Environmental protection importance	0.002	0.003	0.008	0.003	0.02*	0.016^†^	0.004	0.002
Quality importance	0.009	0.005	−0.027*	−0.032**	0.009	0.003	−0.022*	−0.027*
Brand importance	0.005	0.005	−0.02*	−0.023*	0.002	–0.001	–0.016	–0.016
Price importance	0.031**	0.031**	0.023*	0.02*	0.029**	0.026**	0.002	0.001
New and old importance	−0.019*	−0.019*	–0.009	–0.009	–0.001	–0.003	–0.006	–0.006
Age	−	–0.016	−	–0.016	−	–0.009	−	–0.002
Education	−	–0.004	−	0.004	−	0.002	−	0.011
Personal income	−	–0.01	−	–0.014	−	–0.002	−	–0.011
Family income	−	–0.001	−	–0.003	−	−0.01^†^	−	–0.009
Knowledge of remanufactured products	−	0.017	−	0.034*	−	0.022	−	0.013
Experience of remanufactured products	−	0.008	−	0.008	−	−0.026^†^	−	0.017
Knowledge of refurbished products	−	–0.027	−	0.022	−	0.007	−	–0.002
Experience of refurbished products	−	0.008	−	–0.007	−	0.011	−	0.005
*R*2	0.053	0.054	0.063	0.089	0.037	0.042	0.011	0.02
*F*	6.28***	3.039***	7.362***	4.546***	4.593***	2.576***	2.019***	1.739***
Dependent variable	WTP of remanufactured MP4		WTP of refurbished MP4		WTP of remanufactured cartridge		WTP of refurbished cartridge	

^†^*p* < 0.1, *P < 0.05, **P < 0.01, ***P < 0.001; dependent variable: WTP of remanufactured MP4.

Compared with the influencing factors of WTP for remanufactured MP4 and refurbished MP4, consumers’ WTP for refurbished MP4 is also significantly affected by quality and brand attention, but the impact of new and old perceived attention is not significant. Similarly, compared with the WTP for remanufactured cartridges and refurbished cartridges, consumers’ WTP for refurbished cartridges is only significantly affected by the importance of quality. From this point of view, the importance of quality may be one of the important factors affecting the WTP for new products.

The importance of quality is an important factor affecting the WTP of refurbished products. For different products, such as the refurbished MP4, brand and price factors are also significant factors, which may be due to the nature of the product itself.

Based on the analysis above, we understand the influencing factors of consumers’ WTP for remanufactured products as a whole. Generally speaking, the more consumers attach importance to price and environmental protection, the higher their WTP for remanufactured goods. For different characteristics of products, consumers’ WTP will also be different. Therefore, the confirmation of the hypothesis presented in the section “Literature Review” is shown in Table 10.

**TABLE 10:** Test results of the hypothesis for consumers’ WTP.

**Hypothesis**	**Hedonistic product**		**Functional product**	
The more consumers pay attention to environmental protection, the higher the WTP for remanufactured goods.	H3a1	×	H3a2	✓
The more consumers attach importance to quality, the lower their WTP for remanufactured goods.	H3b1	×	H3b2	×
The more consumers attach importance to the brand, the lower the WTP for remanufactured goods.	H3c1	×	H3c2	×
The more consumers pay attention to the price, the higher the WTP for remanufactured goods.	H3d1	✓	H3d2	✓
The more consumers attach importance to the old and the new, the lower the WTP for remanufactured goods.	H3e1	✓	H3e2	×
The higher the consumers’ awareness of remanufacturing, the higher their WTP for remanufactured goods.	H4a1	×	H4a2	×
The higher the level of consumers’ awareness of new products, the higher their WTP for remanufactured products.	H4b1	×	H4b2	×
The richer the consumer’s purchase experience of remanufactured goods, the higher the WTP for remanufactured goods.	H4c1	×	H4c2	✓
The richer the consumer’s purchase experience of remanufactured goods, the higher the WTP for remanufactured goods.	H4d1	×	H4d2	×

## Conclusion and Practical Implications

The writing inspiration of this paper comes from the difficulties encountered by China’s emerging remanufacturing industry. Through the experimental method, this paper investigates the special selection mechanism of consumers for remanufactured products and refurbished products, the influence of refurbished products on the development of remanufactured products, and the influencing factors of consumers’ WTP for remanufactured products, in order to put forward some practical implications on the competition of the remanufacturing industry and the formulation of industrial policy. From the above model, the following conclusions can be drawn:

1. Facing new products, remanufactured products, and refurbished products, most consumers tend to choose new products for products with strong hedonic attributes, and remanufactured products with strong functional properties. For products with strong functional attributes, the impact of refurbished products on remanufactured products is greater than that of products with strong hedonic attributes.
2. For the two types of products, consumers who attach importance to environmental protection are easy to choose remanufactured products with high hedonic attributes, which also have a positive impact on the WTP for functional products. Consumers who attach importance to

price prefer refurbished products instead of remanufactured products. It also shows consumers’ general impression of remanufactured products and new products: environmentally friendly products and cheap products.
3. Consumers who prefer remanufactured products are more likely to be attracted by remanufactured products and have more price-sensitive characteristics than those who prefer new products.
4. Consumers’ WTP for remanufactured products is affected by their subjective attention to new and old, environmental protection and quality, as well as the purchase experience of remanufactured products. In addition to paying attention to the price, for the products with strong hedonic attributes, consumers pay more attention to the new and old products. On the other hand, for the products with strong functional properties, consumers pay more attention to the quality of the products.

On the basis of this, we have the following practical implications for the new Chinese remanufacturing enterprises: (1) When facing the competition of refurbished products, different enterprises should publicize the advantages from different angles according to the attributes of the products. For example, for products with strong functionality, we should highlight the advantages of quality and strive to distinguish them from refurbished products. For products with strong hedonistic attributes, the official certification of manufacturers and the degree of new and old products, as well as the difference between refurbished products, should be emphasized in the market publication. (2) In the process of competition, prices should not be reduced blindly in order to win the market share, which is not conducive to the long-term development of remanufacturing enterprises, and has little effect on promoting the purchase of remanufactured products.

The following are practical implications for relevant government departments: (1) helping increase consumers’ WTP for recycled products, for example, to better educate the public about product reuse; (2) reducing the cost of product recycling, for example, the environmental benefits of subsidies for the collection and processing of waste products; and (3) distinguishing between remanufacturing and refurbishment, helping promote remanufacturing to replace refurbishment, and forming a sound market supervision mechanism to protect the interests of consumers and put an end to shoddy quality.

For OEM production, the following are the practical implications: (1) helping reduce the production cost of new parts and indirectly reduce the cost of remanufacturing (for example, cheaper replacement parts), and (2) making products easy to repair through innovative product design, and improve consumer perception of remanufactured products by emphasizing the production process and quality standards of remanufactured products.

## Data Availability Statement

All datasets generated for this study are included in the article/Supplementary Material.

## Ethics Statement

Ethical review and approval was not required for the study on human participants in accordance with the local legislation and institutional requirements. The patients/participants provided their written informed consent to participate in this study.

## Author Contributions

YC holds the structure of the manuscript. XJ collated the manuscripts. JW revised the manuscript.

## Conflict of Interest

The authors declare that the research was conducted in the absence of any commercial or financial relationships that could be construed as a potential conflict of interest.

## Supplementary Material

DATA SHEET S1 The data of the experiment is contained, as well as the result of the analysis.
